# Etiology and Incidence of Moderate-to-Severe Diarrhea in Young Children in Niger

**DOI:** 10.1093/jpids/piab080

**Published:** 2021-09-01

**Authors:** James A Platts-Mills, Eric R Houpt, Jie Liu, Jixian Zhang, Ousmane Guindo, Nathan Sayinzoga-Makombe, Timothy L McMurry, Sarah Elwood, Céline Langendorf, Rebecca F Grais, Sheila Isanaka

**Affiliations:** 1 Division of Infectious Diseases & International Health, University of Virginia, Charlottesville, Virginia, USA; 2 Epicentre, Niamey, Niger; 3 Department of Public Health Sciences, University of Virginia, Charlottesville, Virginia, USA; 4 Department of Research, Epicentre, Paris, France; 5 Departments of Nutrition and Global Health and Population, Harvard T.H. Chan School of Public Health, Boston, Massachusetts, USA

**Keywords:** children, *Cryptosporidium*, diarrhea, rotavirus, *Shigella*

## Abstract

**Background:**

High-resolution data on the etiology of childhood diarrhea in countries with the highest burden and mortality remain sparse and are needed to inform burden estimates and prioritize interventions.

**Methods:**

We tested stool specimens collected between October 2014 and December 2017 from children under 2 years of age from the per-protocol population of a placebo-controlled clinical trial of a bovine rotavirus pentavalent vaccine (Rotasiil) in Niger. We tested 1729 episodes of moderate-to-severe diarrhea (Vesikari score ≥ 7) using quantitative PCR and estimated pathogen-specific burdens by age, season, severity, and trial intervention arm.

**Results:**

The 4 pathogens with the highest attributable incidence of diarrhea were *Shigella* (7.2 attributable episodes per 100 child-years; 95% confidence interval: 5.2, 9.7), *Cryptosporidium* (6.5; 5.8, 7.2), rotavirus (6.4; 5.9, 6.7), and heat-stabile toxin-producing enterotoxigenic *Escherichia coli* (ST-ETEC) (6.2; 3.1, 7.7). *Cryptosporidium* was the leading etiology of severe diarrhea (Vesikari score ≥ 11) and diarrhea requiring hospitalization. *Shigella* was the leading etiology of diarrhea in children 12-23 months of age but also had a substantial burden in the first year of life, with 60.5% of episodes of severe shigellosis occurring in infants. *Shigella*, *Cryptosporidium*, and ST-ETEC incidence peaked during the warmer and wetter period and coincided with peak all-cause diarrhea incidence.

**Conclusions:**

In this high-burden setting, the leading diarrheal pathogens were *Shigella*, *Cryptosporidium*, rotavirus, and ST-ETEC, and each was disproportionately seen in infants. Vaccine development should target these pathogens, and the impact of vaccine schedule on diarrhea burden in the youngest children will need to be considered.

## Teaser

Using quantitative PCR diagnostics in a phase 3 clinical trial of Rotasiil in Niger, we identified *Shigella*, *Cryptosporidium*, rotavirus, and heat-stabile toxin-producing *Escherichia coli* to be the top 4 leading etiologies of moderate-to-severe diarrhea in children 0-2 years of age.

Although diarrheal mortality has declined globally, there remains a high and disproportionate burden in sub-Saharan Africa, with more than 65% of all diarrheal deaths occurring in this region and about 40% of all diarrheal deaths occurring in Western sub-Saharan Africa alone [1]. The application of quantitative molecular diagnostics has improved the sensitivity and precision of estimates of etiology-specific diarrhea, particularly in settings with high rates of subclinical enteropathogen carriage [2, 3]. Accurate estimates of the etiology-specific incidence of diarrhea are needed from countries with the highest burdens of diarrheal mortality to best guide the prioritization of interventions for these populations of greatest need, including vaccines, point-of-care diagnostic tools, and pathogen-specific therapies.

Niger had the world’s third highest diarrheal mortality in children under 5 years of age in 2016 [1]. We tested stool specimens from Nigerien children under 2 years of age from the per-protocol population of a recent phase 3 clinical trial of a bovine rotavirus pentavalent vaccine (Rotasiil). We performed quantitative polymerase chain reaction (qPCR) for the major agents of diarrhea and applied attribution models previously developed from a large case-control study of moderate-to-severe diarrhea in similar settings [2] to describe the etiology-specific incidence of moderate-to-severe diarrhea [4]. These data can help prioritize the development of interventions to reduce the burden of disease in the populations of highest need.

## MATERIALS AND METHODS

### Study Population, Disease Surveillance, and Sample Selection

We aimed to describe the etiology and incidence of diarrhea in the per-protocol population of a double-blind, placebo-controlled, randomized, phase 3 trial of Rotasiil (Serum Institute of India Pvt Limited, Pune, India) in Madarounfa, Niger (ClinicalTrials.gov Identifier: NCT02145000). The results of the event-driven efficacy trial have been previously described [4]. The per-protocol population included children who received 3 doses of vaccine or placebo without a major protocol violation and excluded children with a laboratory-confirmed rotavirus episode before 28 days after the third vaccine dose. Improved latrine and water sources were defined following World Health Organization (WHO) guidelines [5]. Diarrhea was identified when caregivers presented to healthcare facilities or by home-based surveillance and was defined as 3 or more looser-than-normal stools during a 24-hour period with or without vomiting or presence of blood, with discrete episodes separated by 5 consecutive diarrhea-free days. Dehydration was characterizing using the WHO classification scale [6]. Severity was defined using the 20-point Vesikari clinical scoring system [7], with a score of 7-10 classified as moderate and a score of 11 or more classified as severe.

Stool specimens were collected during or up to 7 days after the end of each episode, transported on freezer packs on the same day, and stored at −80°C. After completion of the study, we randomly selected episodes of moderate (Vesikari score 7-10) to severe (≥11) diarrhea from each of 4 age strata (less than 6, 6-11, 12-17, and 18-23 months), with a goal to test stool specimens from up to approximately 450 episodes per age stratum.

### Microbiologic Studies

Samples were shipped from the study site to the University of Virginia (Virginia, USA) on dry ice and were stored at −80°C before and after shipping. Stool total nucleic acid was then extracted with the QIAamp Fast DNA stool mini kit (Qiagen, Hilden, Germany) using a modified protocol that included bead beating [8]. Phocine herpesvirus and MS2 bacteriophage were spiked into the lysis buffers to monitor extraction and amplification, and extraction banks and no-template controls were included to monitor for contamination. Quantitative PCR (qPCR) testing was performed via a custom TaqMan Array Card (TAC) [9]. Enteropathogen targets on the card included all of the pathogens analyzed by qPCR in the Global Enteric Multicentre Study (GEMS) [2], and diarrheal episodes included in the analysis were required to have valid data for all pathogens included in the final analysis.

### Data Analysis

Because stratified randomization by age group was used to select samples for testing, we used inverse probability weighting to make the selected episodes representative of all episodes of moderate-to-severe diarrhea in the study population. Specifically, we fit a generalized linear model with an outcome of being an episode with valid TAC results for the top 10 etiologies of diarrhea and predictors of age category, year and month of sample collection, calendar week, and child sex. Weights were then calculated as the inverse of the model-predicted probability and standardized by dividing by the mean of the weights.

We have previously demonstrated the importance of the collection of non-diarrheal control stools to more accurately identify etiologic agents in diarrheal stools and have shown that models of the association between pathogen detection and disease from studies which collected both diarrhea and non-diarrheal stools can be applied to subsequent studies where non-diarrheal stools have not been collected, as was the case in the current study [10, 11]. Here, we calculated attributable incidence (AI) as a metric of the burden of etiologic-specific diarrhea for each pathogen. First, we used a model developed from a qPCR reanalysis of cases of non-dysenteric diarrhea and matched controls in GEMS to derive quantity-specific odds ratios for the association between each pathogen and diarrhea [2]. Specifically, using qPCR data from 4077 cases of moderate-to-severe acute watery diarrhea and 1:1 age-, sex-, and village-matched controls in GEMS, we fit a multivariable conditional logistic regression model to describe the association between pathogen quantity and diarrhea while adjusting for the presence of other pathogens. We then calculated a population attributable fraction (AF) for each pathogen, both overall and for each stratum of interest (age, severity, hospitalization, and vaccine arm), for any stratum of *j* episodes as $AF=1\;\frac{\;\;\;\overset{\;j}{\underset{1}{\sum }}\frac{wt_{i}}{OR_{i}}}{\overset{\;j}{\underset{1}{\sum }}wt_{i}}$, where *wt*_*i*_ is the episode-specific inverse probability weight, and *OR*_*i*_ is the episode- and quantity-specific odds ratio derived from the regression model. To estimate the variance for the model-based attribution, the odds ratios were estimated 1000 times using random perturbations of the model coefficients in accordance with their sampling variance-covariance, with estimates taken equally from each of the 7 GEMS site-specific coefficients; 95% confidence intervals (CIs) were derived from the 2.5th and 97.5th quantiles of the AF distribution and the median of the distribution as the point estimate. An AI for each combination of pathogen and stratum was then calculated by dividing by the appropriate person-time. We calculated age-specific attributable episodes as the product of the number of diarrhea episodes identified in each stratum and the weighted AF for that stratum. Pathogens with an overall AF of greater than or equal to one percent were included in the final analysis. As a sensitivity analysis, we recalculated the overall AI estimates using only the models for the African sites from GEMS.

To estimate the association between pathogen-attributable diarrhea and clinical characteristics, a generalized linear mixed-effects model was fit for each characteristic and included the unweighted episode-specific attributable fraction (AFe), where $AFe_{i}=\;1\;1\emptyset R_{i}$, for each of the top 10 overall etiologies of diarrhea, child age, age, a quadratic term for age, and a subject-level random eﬀect. Coefficients were scaled to the AFe range for each pathogen. An AFe for noninfectious diarrhea was defined for each episode as one minus the sum of all pathogen-specific AFes with a lower bound of zero. Finally, to assess the seasonality of attributable diarrhea, we considered an episode etiologic if the pathogen-specific AFe was >0.5. For each pathogen, we then fit a pooled logistic regression model to predict the presence of an etiologic episode in each calendar week, controlling for child age and including the terms $sin\left(\frac{2\pi w}{52}\right)\;\;,\;cosin\left(\frac{2\pi w}{52}\right)\;\;,\;sin\left(\frac{4\pi w}{52}\right)$, and $cosin\left(\frac{4\pi w}{52}\right)\;$ to capture the seasonal variation, where *w* is the calendar week [12]. We used the R survey package v 3.35-1 to incorporate the inverse probability weights in the model. All analyses were performed using R 3.6.0 [13].

## RESULTS

A total of 4128 children were enrolled between 6 and 8 weeks of age from July 31, 2014, to January 1, 2016, and followed per protocol until 2 years of age with 3344 episodes of moderate-to-severe diarrhea identified (Table 1). We selected 1729 episodes by stratified randomization by age group for qPCR testing, of which 1717 (99.3%) had valid results for all included pathogens. The inverse probability weighting rendered the subset of episodes tested by qPCR closely representative of all identified diarrheal episodes, with 30.1% of episodes being severe (Vesikari score ≥ 11) and 8.4% requiring hospitalization. Vomiting was observed in approximately 40% of episodes. The majority of diarrheal episodes occurred in infants, with peak incidence between 6 and 11 months of age.

**Table 1.** Characteristics of Study Population and Episodes of Moderate-to-Severe Diarrhea

Child-Level Characteristics	All Children Included in Etiology Sub-study^a^		
Number of children	4128		
Male sex	2029 (49.2)		
Enrollment weight (kg)	54.3 ± 2.5		
Enrollment length (cm)	4.5 ± 0.7		
Randomized to Rotasiil arm	2096 (50.8)		
Mother completed primary school	271 (6.6)		
Mother can read	1190 (28.9)		
Number of children under 5 in home	2.7 ± 1.3		
No water treatment in home	3753 (90.9)		
Unimproved floor in home	2427 (59.0)		
Unimproved drinking water source	397 (9.7)		
Unimproved latrine	1908 (46.4)		
Episode-Level Characteristics	All Episodes	Episodes With Valid TAC Result (Unweighted)	Episodes With Valid TAC Result (Weighted)
Number of episodes of moderate/severe diarrhea	3344	1715	N/A
Age (wk)	42.5 ± 23.7	49.9 ± 27.8	42.6 ± 23.6
Age 0-5 mo	823 (24.6)	454 (26.4)	422 (24.6)
Age 6-11 mo	1587 (47.5)	451 (26.3)	815 (47.4)
Age 12-17 mo	547 (16.4)	446 (26.0)	281 (16.3)
Age 18-23 m	387 (11.6)	366 (21.3)	200 (11.7)
Vesikari score	9.5 ± 2.5	9.3 ± 2.4	9.5 ± 2.5
Severe (Vesikari >= 11?)	1002 (30.0)	459 (26.7)	517 (30.1)
Duration of diarrhea (d)	3.8 ± 2.3	3.7 ± 2.2	3.8 ± 2.3
Maximum loose stools in 24 h	4.8 ± 1.3	4.7 ± 1.2	4.8 ± 1.3
Vomiting	1386 (41.4)	638 (37.2)	703 (41.0)
Duration of vomiting (days)	2.1 ± 1.6	2 ± 1.6	2.1 ± 1.6
Maximum vomiting episodes in 24 h	2.2 ± 1.5	2.2 ± 1.4	2.2 ± 1.4
Objective fever (>= 38.0°C)	620 (18.5)	325 (18.9)	331 (19.3)
Severe dehydration	49 (1.5)	27 (1.6)	31 (1.8)
Some dehydration	238 (7.1)	110 (6.4)	128 (7.4)
No dehydration	3057 (91.4)	1580 (92.0)	1558 (90.7)
Hospitalized	277 (8.6)	127 (7.7)	139 (8.4)

Abbreviation: TAC, TaqMan Array Card.

^a^Data shown are N (%) for dichotomous variables and mean ± standard deviation for continuous variables.

The 4 pathogens with the highest AI of moderate-to-severe diarrhea in children 0 to 23 months of age were *Shigella* (7.2 attributable episodes per 100 child-years; 95% CI: 5.2, 9.7), *Cryptosporidium* (6.5; 5.8, 7.2), rotavirus (6.4; 5.9, 6.7), and heat-stabile toxin-producing enterotoxigenic *Escherichia coli* (ST-ETEC) (6.2; 3.1, 7.7) (Table 2). Out of an all-cause moderate-to-severe diarrhea incidence of 43.4 episodes per 100 child-years, 41.3 (95.2%) were attributable to the 11 pathogens included in the analysis. Rotavirus was the only viral pathogen among the top 4 etiologies identified, and more than half of the total AI (24.8 episodes per 100 child-years; 60.0%) was attributable to a bacterial or protozoal pathogen. As defined by the parent trial protocol, children with rotavirus diarrhea identified by enzyme immunoassay within 28 days of the third and final doses of vaccine were excluded from the per-protocol group. Limiting the analysis to the period starting 28 days after the third dose of vaccine did not change the order of importance of the top etiologies. Alternatively, calculating the overall AI using the GEMS models from African sites only did not change the hierarchy of the top 5 pathogens, and the estimate changed by more than 5% only for ST-ETEC, for which the estimate decreased by −11.0% (5.6 attributable episodes per 100 child-years, 95% CI: 2.5, 7.5).

**Table 2.** Attributable Incidence of All-Cause and Etiology-Specific Moderate-to-Severe Diarrhea, Per 100 Child-Years

	Any cause	*Shigella*	*Cryptosporidium*	Rotavirus	ST-ETEC	Adenovirus 40/41	*Campylobacter jejuni/coli*	Norovirus GII	Astrovirus	Sapovirus	Typical EPEC	*Salmonella*
Overall	43.4	7.2 (5.2, 9.7)	6.5 (5.8, 7.2)	6.4 (5.9, 6.7)	6.2 (3.1, 7.7)	4.0 (3.3, 4.5)	3.2 (0.0, 7.5)	2.4 (1.2, 3.3)	2.1 (0.9, 2.8)	1.6 (0.0, 3.4)	1.2 (0.0, 4.3)	0.5 (0.4, 0.6)
Overall^a^	43.3	8.0 (5.6, 10.9)	7.1 (6.2, 7.8)	7.0 (6.5, 7.3)	7.0 (3.2, 8.7)	4.2 (3.5, 4.8)	3.4 (0.0, 8.8)	2.3 (1.0, 3.1)	2.2 (1.0, 2.9)	1.7 (0.0, 3.8)	1.3 (0.0, 4.6)	0.5 (0.4, 0.6)
Age												
0-5 mo^b^	54.6	2.5 (1.7, 3.3)	5.9 (5.2, 6.5)	6.6 (6.0, 6.9)	4.7 (2.1, 5.9)	4.8 (3.9, 5.5)	2.2 (0.0, 5.1)	4.4 (2.2, 6.2)	3.0 (1.3, 4.1)	0.9 (0.0, 2.0)	1.2 (0.0, 4.4)	0.4 (0.3, 0.4)
6-11 mo	76.7	12.8 (9.0, 17.3)	13.6 (12.1, 14.9)	12.7 (11.8, 13.2)	11.9 (5.7, 14.8)	8.4 (7.0, 9.4)	6.6 (0.0, 16.1)	4.7 (1.9, 6.5)	4.3 (1.9, 5.8)	3.5 (0.0, 7.6)	2.4 (0.0, 9.4)	1.0 (0.8, 1.1)
12-17 mo	26.4	6.0 (4.2, 8.3)	3.5 (3.1, 3.9)	4.9 (4.5, 5.1)	4.9 (2.2, 6.1)	2.5 (2.0, 2.8)	2.1 (0.0, 5.1)	0.7 (0.3, 1.0)	0.9 (0.4, 1.2)	0.9 (0.0, 2.1)	0.5 (0.0, 2.0)	0.2 (0.1, 0.2)
18-23 mo	18.8	6.0 (4.2, 8.2)	2.8 (2.4, 3.1)	1.5 (1.4, 1.6)	3.1 (1.4, 3.9)	0.7 (0.5, 0.8)	1.5 (0.0, 3.9)	0.3 (0.1, 0.5)	0.3 (0.1, 0.5)	0.5 (0.0, 1.3)	0.4 (0.0, 1.4)	0.5 (0.4, 0.6)
Severity												
Severe	13	1.7 (1.2, 2.3)	2.6 (2.3, 2.9)	2.4 (2.2, 2.5)	1.9 (0.9, 2.4)	1.4 (1.2, 1.7)	1.0 (0.0, 2.4)	0.8 (0.4, 1.0)	0.6 (0.2, 0.8)	0.5 (0.0, 1.1)	0.4 (0.0, 1.5)	0.2 (0.1, 0.2)
<12 mo	22.9	2.2 (1.5, 3.0)	4.7 (4.2, 5.2)	4.0 (3.7, 4.2)	3.3 (1.5, 4.0)	2.7 (2.3, 3.1)	1.8 (0.0, 4.1)	1.5 (0.7, 2.1)	1.1 (0.5, 1.5)	0.9 (0.0, 1.9)	0.7 (0.0, 2.7)	0.2 (0.2, 0.3)
12-23 mo	4.5	1.0 (0.7, 1.4)	0.8 (0.7, 0.9)	1.3 (1.2, 1.3)	0.8 (0.4, 1.0)	0.4 (0.3, 0.5)	0.3 (0.0, 0.9)	0.1 (0.1, 0.2)	0.2 (0.1, 0.2)	0.2 (0.0, 0.3)	0.1 (0.0, 0.2)	0.0 (0.0, 0.0)
Moderate	30.4	5.5 (4.0, 7.4)	3.9 (3.5, 4.3)	4.0 (3.7, 4.2)	4.3 (2.2, 5.4)	2.6 (2.1, 2.9)	2.2 (0.0, 5.5)	1.7 (0.7, 2.3)	1.5 (0.7, 2.0)	1.0 (0.0, 2.3)	0.8 (0.0, 3.0)	0.4 (0.3, 0.4)
Hospitalization												
Yes	3.6	0.5 (0.3, 0.6)	0.9 (0.8, 0.9)	0.4 (0.4, 0.5)	0.4 (0.2, 0.5)	0.4 (0.4, 0.5)	0.2 (0.0, 0.5)	0.2 (0.1, 0.2)	0.2 (0.1, 0.2)	0.2 (0.0, 0.3)	0.1 (0.0, 0.3)	0.2 (0.1, 0.2)
No	38	6.5 (4.5, 8.7)	5.4 (4.7, 5.9)	5.8 (5.3, 6.0)	5.6 (2.4, 7.1)	3.4 (2.8, 3.9)	2.8 (0.0, 6.9)	2.2 (1.0, 3.0)	1.9 (0.8, 2.5)	1.3 (0.0, 2.8)	1.1 (0.0, 3.9)	0.3 (0.3, 0.4)
Received Rotasiil												
Overall	33.9	6.5 (4.6, 8.7)	5.2 (4.6, 5.7)	4.6 (4.2, 4.8)	5.3 (2.5, 6.6)	3.3 (2.8, 3.8)	2.2 (0.0, 5.9)	1.8 (0.8, 2.5)	1.2 (0.5, 1.6)	1.3 (0.0, 2.9)	0.9 (0.0, 3.5)	0.5 (0.4, 0.6)
<12 mo	49	7.4 (5.3, 9.7)	7.8 (7.0, 8.6)	6.6 (6.0, 6.9)	6.8 (3.0, 8.5)	5.6 (4.6, 6.3)	3.0 (0.0, 7.8)	3.3 (1.4, 4.6)	1.8 (0.7, 2.5)	2.1 (0.0, 4.6)	1.6 (0.0, 6.0)	0.6 (0.5, 0.7)
12-23 mo	20.8	5.1 (3.6, 6.9)	2.6 (2.3, 2.9)	3.5 (3.2, 3.7)	4.2 (1.9, 5.3)	1.8 (1.5, 2.1)	1.8 (0.0, 4.3)	0.7 (0.3, 0.9)	0.8 (0.3, 1.1)	0.6 (0.0, 1.4)	0.5 (0.0, 1.7)	0.2 (0.1, 0.2)
Severe	11.7	1.7 (1.2, 2.2)	2.4 (2.1, 2.6)	1.5 (1.4, 1.6)	1.7 (0.9, 2.1)	1.5 (1.2, 1.7)	0.8 (0.0, 2.0)	0.9 (0.4, 1.3)	0.4 (0.2, 0.6)	0.5 (0.0, 1.0)	0.4 (0.0, 1.4)	0.2 (0.2, 0.2)
<12 mo	21.1	2.4 (1.8, 3.2)	4.1 (3.6, 4.5)	2.4 (2.1, 2.5)	3.0 (1.4, 3.6)	2.9 (2.4, 3.3)	1.4 (0.0, 3.6)	1.9 (0.7, 2.5)	0.8 (0.3, 1.1)	0.9 (0.0, 2.0)	0.9 (0.0, 2.9)	0.4 (0.3, 0.4)
12-23 mo	3.6	0.9 (0.6, 1.2)	0.8 (0.8, 0.9)	0.8 (0.8, 0.9)	0.8 (0.3, 0.9)	0.3 (0.3, 0.3)	0.3 (0.0, 0.8)	0.2 (0.1, 0.3)	0.2 (0.1, 0.2)	0.0 (0.0, 0.1)	0.1 (0.0, 0.2)	0.0 (0.0, 0.0)
Moderate	22.2	4.8 (3.3, 6.4)	2.8 (2.5, 3.1)	3.1 (2.8, 3.2)	3.5 (1.6, 4.4)	1.9 (1.6, 2.1)	1.5 (0.0, 3.7)	0.9 (0.4, 1.2)	0.7 (0.3, 1.0)	0.8 (0.0, 1.9)	0.6 (0.0, 2.1)	0.3 (0.2, 0.4)
Received placebo												
Overall	53.2	7.9 (5.4, 10.9)	8.0 (7.0, 8.8)	8.3 (7.6, 8.6)	7.3 (3.4, 9.1)	4.7 (3.9, 5.4)	4.0 (0.0, 9.8)	3.0 (1.3, 4.2)	3.0 (1.1, 4.0)	1.7 (0.0, 3.8)	1.2 (0.0, 5.1)	0.5 (0.4, 0.6)
<12 mo	86.3	9.6 (6.8, 13.1)	13.1 (11.6, 14.4)	13.8 (12.5, 14.3)	11.0 (5.0, 13.6)	8.2 (6.8, 9.3)	5.9 (0.0, 15.1)	5.9 (2.8, 8.3)	5.8 (2.3, 7.8)	2.7 (0.0, 6.0)	2.4 (0.0, 9.2)	0.9 (0.7, 1.0)
12-23 mo	24.5	5.2 (3.6, 7.3)	3.5 (3.1, 3.8)	4.8 (4.5, 5.0)	4.1 (1.9, 5.1)	2.5 (2.0, 2.8)	1.8 (0.0, 4.7)	0.5 (0.2, 0.7)	0.7 (0.3, 1.0)	1.0 (0.0, 2.3)	0.4 (0.0, 1.6)	0.1 (0.1, 0.1)
Severe	14.3	1.7 (1.2, 2.4)	2.9 (2.5, 3.2)	3.3 (3.1, 3.4)	2.0 (0.8, 2.6)	1.4 (1.2, 1.7)	1.1 (0.0, 2.7)	0.6 (0.3, 0.8)	0.7 (0.3, 1.0)	0.5 (0.0, 1.1)	0.4 (0.0, 1.4)	0.1 (0.1, 0.1)
<12 mo	24.7	2.0 (1.4, 2.8)	5.3 (4.7, 5.8)	5.7 (5.2, 5.9)	3.5 (1.7, 4.5)	2.6 (2.1, 3.0)	1.8 (0.0, 4.6)	1.2 (0.5, 1.7)	1.5 (0.6, 1.9)	0.8 (0.0, 1.8)	0.7 (0.0, 2.7)	0.1 (0.1, 0.2)
12-23 mo	5.3	1.1 (0.8, 1.6)	0.8 (0.7, 0.9)	1.7 (1.6, 1.7)	0.8 (0.4, 1.0)	0.5 (0.4, 0.6)	0.4 (0.0, 0.9)	0.1 (0.0, 0.1)	0.2 (0.1, 0.2)	0.3 (0.0, 0.6)	0.0 (0.0, 0.2)	0.0 (0.0, 0.0)
Moderate	38.8	6.1 (4.4, 8.3)	5.0 (4.4, 5.5)	4.9 (4.5, 5.1)	5.2 (2.5, 6.5)	3.3 (2.7, 3.8)	2.8 (0.0, 6.8)	2.5 (1.2, 3.4)	2.3 (0.8, 3.1)	1.3 (0.0, 2.7)	0.9 (0.0, 3.4)	0.4 (0.3, 0.5)

Abbreviations: ST-ETEC, heat-stabile toxin-producing enterotoxigenic *Escherichia coli;* EPEC, enteropathogenic *E. coli*.

^a^Starting 28 days after the third dose of vaccine.

^b^Subjects with rotavirus diarrhea occurring before 28 days after the third dose of vaccine were excluded from the per-protocol group.

Pathogen-specific AI varied by age, severity, the need for hospitalization, and trial intervention arm. All pathogens included in the analysis had a higher AI in infants. *Cryptosporidium* was the leading etiology of severe diarrhea in children under 12 months of age as well as diarrhea requiring hospitalization. Among vaccinated children, *Cryptosporidium, Shigella*, ST-ETEC, and adenovirus 40/41 had an AI of severe diarrhea greater than or equal to that of rotavirus, whereas rotavirus was the leading etiology of severe diarrhea in children who received placebo. *Shigella* was not only the leading etiology of diarrhea in children 12-23 months of age, but also had a substantial burden in the first year of life, such that 54.9% of episodes of shigellosis, and 60.5% of episodes of severe shigellosis occurred in infants (Table 3). More than 75% of severe episodes attributable to rotavirus, *Cryptosporidium*, and ST-ETEC occurred in the first year. The impact of rotavirus vaccine was clearly evident, with a reduction in the incidence of severe rotavirus diarrhea in infants from 5.7 attributable episodes per 100 child-years (95% CI: 5.2, 5.9) in the placebo group to 2.4 attributable episodes (95% CI: 2.1, 2.5) in the vaccine group, consistent with the efficacy reported in the primary trial analysis [4]. The incidence of severe *Cryptosporidium* was also reduced in infants randomized to rotavirus vaccine.

**Table 3.** Distribution of Attributable Episodes by Age for Top 4 Etiologies of Diarrhea

Age	*Shigella*-Attributable Episodes (Cumulative %)	Severe *Shigella*- Attributable Episodes (Cumulative %)	*Cryptosporidium*- Attributable Episodes (Cumulative %)	Severe *Cryptosporidium-*Attributable Episodes (Cumulative %)	Rotavirus-Attributable Episodes (Cumulative %)	Severe Rotavirus-Attributable Episodes (Cumulative %)	ST-ETEC*-*Attributable Episodes (Cumulative %)	Severe ST-ETEC-Attributable Episodes (Cumulative %)
1-3 mo^a^	0.8 (0.1)	0.2 (0.2)	7.9 (1.6)	2.2 (1.1)	14.5 (2.9)	6.3 (3.4)	1.4 (0.3)	0 (0)
3-5 mo	35.8 (6.7)	12.1 (9.7)	81.9 (17.7)	39 (20.2)	84.5 (20)	38.4 (24.3)	69.3 (14.7)	27.7 (18.8)
6-8 mo	139.3 (32.1)	35.1 (37.2)	179.8 (53.3)	84.5 (61.7)	163.1 (53.1)	60.5 (57.1)	153.7 (46.5)	58.2 (58.3)
9-11 mo	124.9 (54.9)	29.7 (60.5)	105.6 (74.2)	44.4 (83.5)	100 (73.3)	38.7 (78.1)	93.1 (65.8)	31.3 (79.5)
12-14 mo	69.8 (67.7)	18.4 (75)	42.8 (82.6)	16.3 (91.5)	66 (86.7)	20.9 (89.4)	58.1 (77.9)	16.7 (90.8)
15-17 mo	53.2 (77.4)	9.7 (82.6)	30.2 (88.6)	6.9 (94.8)	34.5 (93.6)	14.5 (97.3)	43.2 (86.8)	5.6 (94.6)
18-20 mo	59.7 (88.3)	9.6 (90.1)	29.3 (94.4)	6.2 (97.9)	17.5 (97.2)	4 (99.5)	32.5 (93.6)	2.2 (96.1)
21-23 mo	64.2 (100)	12.6 (100)	28.5 (100)	4.3 (100)	13.9 (100)	1 (100)	31 (100)	5.7 (100)

Abbreviation: ST-ETEC, heat-stabile toxin-producing enterotoxigenic *Escherichia coli.*

^a^Children were enrolled between 6 and 8 wk of age.

Specific pathogens were associated with unique clinical profiles (Table 4). Diarrhea attributable to rotavirus and adenovirus 40/41 were more likely to be accompanied by vomiting and dehydration, whereas *Cryptosporidium* was associated with a prolonged duration, a high Vesikari score, and hospitalization. *Shigella* diarrhea was also prolonged, whereas *Salmonella* was associated with fever, dehydration, and hospitalization.

**Table 4.** Clinical Features Associated With Etiology-Specific Diarrhea

	Objective Fever (n = 325)	Prolonged Duration (≥7 days) (n = 147)	High Frequency (>= 6 Loose Stools in 24 h) (n = 374)	Vomiting (n = 636)	Severe (Vesikari Score >= 11) (*n* = 458)	Any Dehydration (*n* = 136)	Hospitalization^a^ (*n* = 127)
	Prevalence ratio (95% CI)						
Bacteria							
*Campylobacter jejuni/coli*	1.06 (0.73-1.56)	0.66 (0.37-1.19)	0.84 (0.59-1.21)	0.85 (0.65-1.12)	0.91 (0.66-1.26)	0.75 (0.42-1.36)	0.36 (0.18-0.70)
ST-ETEC	0.68 (0.46-0.99)	0.82 (0.46-1.45)	1.16 (0.84-1.62)	1.04 (0.81-1.34)	1.02 (0.76-1.37)	1.30 (0.78-2.18)	0.41 (0.20-0.82)
*Salmonella*	3.58 (1.70-7.52)	3.04 (0.89-10.36)	0.32 (0.06-1.64)	0.99 (0.38-2.58)	1.92 (0.79-4.64)	3.82 (1.04-14.05)	6.25 (2.39-16.34)
*Shigella*	0.70 (0.48-1.03)	1.68 (0.99-2.87)	1.47 (1.05-2.05)	0.74 (0.55-1.00)	0.93 (0.67-1.29)	1.12 (0.65-1.95)	0.95 (0.52-1.74)
Typical EPEC	0.96 (0.63-1.47)	1.13 (0.63-2.04)	1.09 (0.75-1.60)	1.17 (0.88-1.56)	1.23 (0.88-1.71)	2.21 (1.26-3.90)	0.65 (0.31-1.35)
Viruses							
Adenovirus 40/41	0.83 (0.50-1.39)	1.28 (0.64-2.55)	1.21 (0.78-1.87)	1.44 (1.05-1.97)	1.41 (0.98-2.05)	1.95 (1.05-3.60)	1.55 (0.73-3.25)
Astrovirus	0.82 (0.44-1.53)	0.63 (0.25-1.58)	1.06 (0.62-1.79)	0.91 (0.60-1.36)	0.80 (0.49-1.31)	0.84 (0.34-2.06)	0.78 (0.29-2.11)
Norovirus GII	0.67 (0.36-1.23)	0.45 (0.18-1.14)	0.77 (0.45-1.32)	1.29 (0.90-1.85)	1.06 (0.67-1.65)	0.34 (0.11-1.05)	0.50 (0.17-1.44)
Rotavirus	0.80 (0.56-1.15)	0.59 (0.31-1.13)	0.98 (0.70-1.37)	1.73 (1.39-2.14)	1.60 (1.23-2.07)	1.65 (1.03-2.63)	0.90 (0.50-1.62)
Sapovirus	0.70 (0.37-1.30)	0.89 (0.35-2.25)	1.00 (0.57-1.76)	1.06 (0.70-1.61)	1.08 (0.67-1.74)	1.38 (0.61-3.10)	1.10 (0.45-2.71)
Protozoa							
*Cryptosporidium*	0.86 (0.58-1.27)	3.08 (1.92-4.96)	0.97 (0.67-1.40)	1.44 (1.11-1.87)	1.98 (1.49-2.64)	1.74 (1.02-2.98)	2.84 (1.66-4.84)
No etiology identified	1.32 (0.96-1.82)	0.87 (0.53-1.42)	0.77 (0.56-1.05)	0.67 (0.52-0.86)	0.57 (0.42-0.77)	0.40 (0.22-0.73)	1.10 (0.65-1.86)

Abbreviations: CI, confidence interval; ST-ETEC, heat-stabile toxin-producing enterotoxigenic *Escherichia coli*; EPEC, enteropathogenic *E. coli*.

^a^Data on hospitalization are available for 1657/1717 episodes (96.5%).

**Figure 1.**
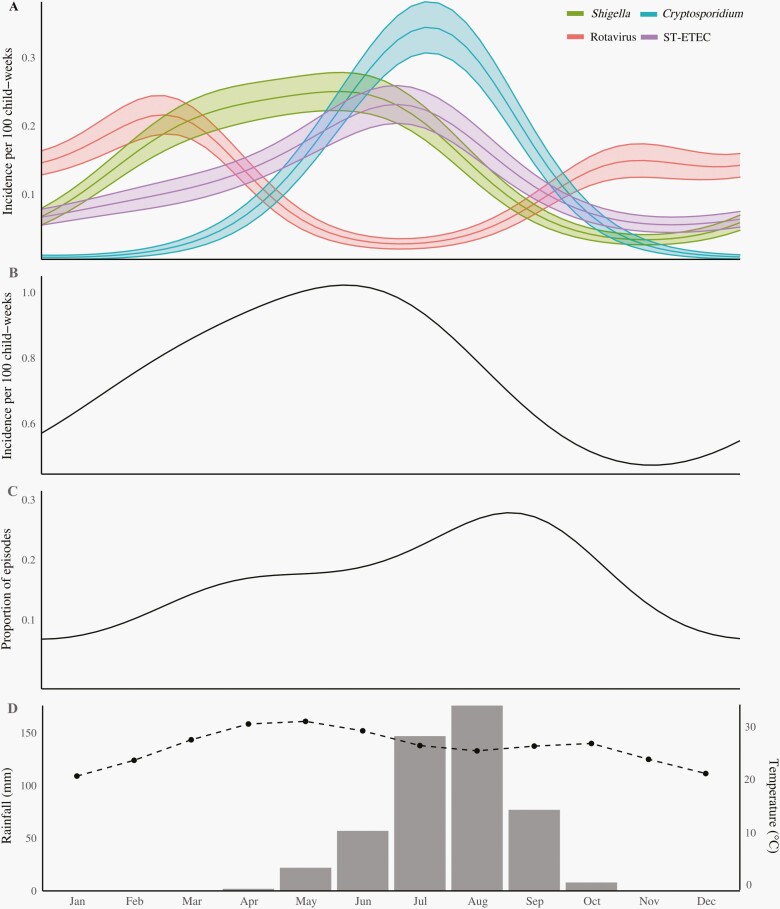
Seasonality of etiology-specific diarrhea in Niger. Panels show (A) model-predicted seasonal incidence of diarrhea due to the top 4 identified etiologies; (B) model-predicted seasonal incidence of all-cause diarrhea; (C) model-predicted seasonal proportion of episodes with more than one etiologic pathogen identified; and (D) average seasonal rainfall (bars) and temperature (dotted line) in Madarounfa, Niger. Abbreviation: ST-ETEC, heat-stabile toxin-producing enterotoxigenic *Escherichia coli*.

The seasonality of the top 4 etiologies of diarrhea was striking (Figure 1), with rotavirus peaking during the relatively colder and drier period of the year, and *Shigella*, *Cryptosporidium*, and ST-ETEC peaking during the warmer and wetter period. This latter period coincided with the peak in all-cause diarrhea incidence, as well as a peak in the proportion of episodes that had more than one attributable pathogen.

## Discussion

In this analysis of the etiology of moderate-to-severe diarrhea in Niger, a country in the highest stratum of diarrhea mortality, we found a particularly high burden of rotavirus, *Shigella*, *Cryptosporidium*, and ST-ETEC. The overall incidence of severe diarrhea was roughly comparable to other rotavirus vaccine efficacy trials in Africa [14–16], and the impact of rotavirus vaccine was clear and consistent with that observed in the primary analysis [4]. These findings are mostly consistent with previous studies of the etiology and incidence of diarrhea in the first 2 years of life in low-resource settings [2, 3], in particular in that the top 6 causes of diarrhea (ie, the 4 previously mentioned as well as adenovirus 40/41 and *Campylobacter jejuni/coli*) were the same. However, the burden of bacterial and protozoal pathogens, in particularly *Shigella* and *Cryptosporidium*, was particularly high and especially among infants.

Recent etiologic studies have highlighted a particularly high burden of *Shigella* identified using molecular methods, but disproportionately occurring in the second year of life [2, 3]. In a cohort study of community diarrhea in 8 settings in South America, Africa, and Asia, approximately three-quarters of shigellosis incidence in children under 2 years of age occurred in the second year of life [3]. In this study, by contrast, more than half of *Shigella*-attributable diarrhea occurred in infants, and the incidence of severe shigellosis in infants was approximately double that of children 12-23 months of age. The current working goal for a *Shigella* vaccine to provide full protection by 12 months of age [17] would not provide direct protection against severe shigellosis in infants. While it is possible that indirect (herd) protection will have sufficient effectiveness for this strategy to be successful, this will need to be evaluated in clinical trials and post-introduction impact studies [18].

This study supports other evidence of the high burden and severity of cryptosporidiosis in sub-Saharan Africa [2, 3]. In this study, it was the leading etiology of severe diarrhea in infants, who received Rotasiil, and the leading etiology of diarrhea requiring hospitalization in the overall study population. The HIV status of the participants in this study was not assessed but would be expected to be below in this age group. Surprisingly, the incidence of severe cryptosporidiosis was lower in children randomized to rotavirus vaccine, consistent with a previously described reduction in severe cryptosporidiosis in children randomized to rotavirus vaccine in a clinical trial in India [11]. While these could be spurious findings, they could alternatively suggest a nonspecific benefit of rotavirus vaccination against this illness [19]. Other high-burden pathogens in this population included ST-ETEC and adenovirus 40/41, broadly consistent with studies from these settings [2, 3]. Interestingly, *Salmonella* was a strong predictor of hospitalization, a finding that has not been consistently observed in other studies of diarrhea [3] and might suggest a high proportion of episodes accompanied by invasive disease. Episodes of diarrhea without an attributable etiology were more likely to be accompanied by fever but were milder and less likely to be accompanied by vomiting and dehydration. This might suggest that these episodes were due to a non-gastrointestinal pathogen, eg, malaria [20].

The combined high burden of diarrhea due to bacterial and protozoal pathogens in this study of predominantly watery diarrhea raises the question as to whether current guidelines which defer antimicrobial therapy in the absence of bloody diarrhea are sufficient in this setting [6] and suggests the potential value of clinical prediction tools or point-of-care diagnostics to better target antimicrobial therapy [21]. Evidence from a multisite clinical trial is forthcoming about the role of azithromycin in treating watery diarrhea [22] which may help further direct the value of point-of-care identification of bacterial enteropathogens in the absence of dysentery. Point-of-care diagnostics [23] and novel therapeutics [24] for *Cryptosporidium* are areas of active areas of investigation and on the basis of these findings should be prioritized.

Each of the top 4 pathogens demonstrated significant seasonal variation in incidence. Rotavirus diarrhea peaked during the dry and relatively cooler season, consistent with the global epidemiology of this pathogen [25, 26]. Diarrhea attributable to *Shigella, Cryptosporidium,* and ST-ETEC peaked during the wetter and relatively warmer season, during which all-cause diarrhea incidence also peaked. Driven by the co-seasonality of these highest-burden pathogens, the proportion of diarrhea episodes with more than one etiology identified also peaked during this season. There is some evidence that rotavirus vaccine efficacy estimates are artificially lowered by misattribution of rotavirus as the etiology of diarrhea due to a second pathogen [11]. However, the seasonal coincidence of diarrhea due to these other high-burden pathogens observed here suggests that misattribution may be a more significant problem for these other etiologies. Diagnostics to more broadly interrogate and exclude or at least account for the presence of co-etiologic pathogens may be necessary in efficacy trials of new vaccines in development targeting these pathogens, in addition to having significant clinical value.

This study has several important limitations. First, although dysentery was not explicitly excluded from this study, the presence of blood in stool was not in itself sufficient to meet the study definition. Thus, some episodes of dysentery may have been excluded. Similarly, the use of a Vesikari score, in which vomiting plays a prominent role, as the criteria for identifying moderate-to-severe diarrhea may have favored viral etiologies. In total, this may mean that the observed high burden of severe bacterial and protozoal diarrhea is still an underestimate. Second, as per the parent trial protocol, rotavirus diarrhea was explicitly excluded from the per-protocol group through 28 days after the last dose of vaccine. Estimates of etiology-specific incidence excluding this time frame, however, did not significantly alter the findings. Finally, non-diarrheal stools were not collected in this study. While we have previously demonstrated that models developed from other studies with carefully collected non-diarrheal stools can be used to attribute etiology in studies without non-diarrheal stools [10, 11], this assumes that previously assayed non-diarrheal samples are representative of this setting, which may not be the case. These attribution methods may also be particularly conservative for pathogens with a high burden of subclinical carriage, leading to relatively imprecise estimates for pathogens such as *Campylobacter* and norovirus.

Conclusion

This study adds critical data to the current evidence on the etiology of diarrhea using molecular methods in sub-Saharan Africa and raises important questions for the diagnosis, treatment, and prevention of enteric pathogens in this setting. In particular, this study suggests that in addition to rotavirus, *Shigella*, *Cryptosporidium*, and ST-ETEC are high priority pathogens given their high burden in this high mortality setting.

## Notes

***Acknowledgment.*** We thank Matthew Hitchings for his statistical support.

***Financial support.*** J.A.P., E.R.H., J.L., J.Z., T.L.M., and S.E. were supported by the Bill & Melinda Gates Foundation (award number OPP1179069 to E.R.H).

***Potential conflicts of interest.*** All authors: No reported conflicts.
